# Rheological Behaviour of Cementitious Materials Incorporating Solid–Solid Phase Change Materials

**DOI:** 10.3390/ma15010020

**Published:** 2021-12-21

**Authors:** Lionel Plancher, Alexandre Pierre, Giao T. M. Nguyen, Ronan L. Hébert, Béatrice A. Ledésert, Patrick Di Martino, Yannick Mélinge

**Affiliations:** 1 Laboratoire L2MGC, CY Cergy Paris University, 95031 Neuville sur Oise, France; lionel.plancher@cyu.fr (L.P.); yannick.melinge@culture.gouv.fr (Y.M.); 2 Laboratoire LPPI, CY Cergy Paris University, 95031 Neuville sur Oise, France; tran-minh-giao.nguyen@cyu.fr; 3 Laboratoire GEC, CY Cergy Paris University, 95031 Neuville sur Oise, France; Ronan.hebert@cyu.fr (R.L.H.); beatrice.ledesert@cyu.fr (B.A.L.); 4 Laboratoire ERRMECe, CY Cergy Paris University, 95031 Neuville sur Oise, France; patrick.di-martino@cyu.fr; 5 Fédération Institut des Matériaux I-Mat (FD4122), CY Cergy Paris Université, 95031 Neuville sur Oise, France

**Keywords:** phase change materials, cementitious materials, rheology

## Abstract

Nowadays, thermal regulation of the indoor environment is mandatory to reduce greenhouse gas emissions. The incorporation of Phase Change Materials (PCMs) and especially solid–solid PCMs (s/s PCMs) into building materials can be a major step forward in reducing energy consumption. Such materials are used for their high latent heat to save and release heat during phase change. To integrate these products in the fabrication of cementitious materials, it is essential to predict their influence on the rheological behaviour of construction materials. In this work, rheological measurements were carried out on composite suspensions made of cement or mortar plus s/s PCMs. Results showed that the fitting of the Herschel–Bulkley model with a constant value of flow exponent was reliable. The s/s PCMs influenced the consistency and the yield strength values, with the yield strength value being only slightly affected. The adaptation of an existing viscosity model is proposed to predict the consistency value of suspensions. Finally, an innovative approach to predict the flow behaviour is proposed and we highlight the research needs to mainstream the use of s/s PCMs in construction materials.

## 1. Introduction

The reduction of energy consumption and greenhouse gas (GHG) emissions is an important issue. While worldwide demand for energy keeps increasing, international commitments have been ratified to limit GHG emissions [1,2]. Building heating and air conditioning require intensive use of resources and energy [3]. In 2016, thermal regulation of the indoor environment was responsible for 25% of the total energy consumption in France [4].

Enhancing the thermal efficiency of buildings is a proper way to reduce the energy consumption of indoor thermal regulation and some government plans are already engaged in this regard (“Energy renovation plan for buildings” for example in France). The use of Phase Change Materials (PCMs) use in inhabitation is a novel way to reduce energy consumption for several decades [5,6,7,8,9]. The high latent heat that characterises those materials is a real contribution to saving and releasing energy during reversible phase change. Most of the time these transitions consist in melting and crystallisation of the materials. Indeed, solid/liquid PCMs (s/l PCMs) in a solid state at a temperature below a melting one undergo an endothermic transition that leads to a liquid state when the temperature increases, allowing the storage of thermal energy in the materials. Conversely, when the temperature decreases, they change from a liquid state to a solid one through an exothermic transition. Consequently, thermal energy stored is released. In order to be used in building applications, those PCMs require to be embedded in solid matrix in order to avoid fluid leakage. Tyagi et al., [10] reviewed ten encapsulation technologies used to contain s/l PCMs and their applications in building materials [11,12,13,14,15,16,17,18,19]. Micro or macro-encapsulated PCMs developed by BASF, Climator, Cristopia, Dupont de Nemours, Rubitherm and Winco technologies are commercialized nowadays. Despite some convincing efficiency, s/l PCMs are not mainly incorporated because of several drawbacks, mainly the extra cost of encapsulation as well as the risk of PCM leakage issue. In order to overcome these disadvantages, and in particular, to prevent leakage, solid–solid PCMs (s/s PCMs) have recently become a subject of great interest [20,21,22,23,24,25]. s/s PCMs undergo a microstructural phase change from crystalline to amorphous while remaining at a solid state. Details of the structural changes can be found in Harlé et al. [25]. The same authors in [25] reported cross-linked poly (ether urethane) (PUX)s as solid–solid phase change materials that can be ground into powder and incorporated into hydraulic-based construction materials.

Incorporating such solutions directly on site construction is challenging and requires a better knowledge of their influence on cementitious material flow. The rheology of grouts, mortars and concretes is of major interest for many applications in the construction industry [26,27]. Their rheological behaviour is mainly due to the interactions between mineral particles and chemical admixtures. These admixtures are currently used as additives leading to the desired workability and rheology of fresh concrete depending on the application such as pumping, deposition, 3D printing, etc. For example, to prevent bleeding, viscosity modifying admixtures (VMA) are used, such as either cellulose or welan gum [28,29]. These admixtures increase the water-retaining properties of the freshly mixed concrete and can be used for pre-cast processes such as extrusion, to prevent liquid drainage during production [30]. Superplasticizers such as polycarboxylate polymers (PCP) lead to a better fluidity of the suspension and a decrease in the yield stress [31,32,33].

Materials generally used in building applications exhibit elasto-viscoplastic rheological behaviour under fresh state. The suspensions can flow as soon as the stress applied to the system exceeds the limit shear stress of the network of interacting particles [6]. The state of the art concerning the influence of PCMs on the rheological behaviour of cementitious materials is not abundant. Some studies showed that adding micro-encapsulated s/l PCMs in the mix-design results in a significant change in flow properties [5,34,35]. This study aims to clarify the role of an s/s PCM on cementitious materials flow properties.

Firstly, the used materials and the used s/s PCMs in this study are described. In addition, the influence of the used s/s PCMs and their mix proportion on the yield stress and consistency of the cement and mortar suspensions are investigated. To achieve this objective, mechanical assumptions from existing physical theory and polymer rheological law are used. Finally, based on the existing model, the underlying mechanism of the effect of s/s PCMs in cement suspensions rheology is highlighted.

## 2. Materials and Methods

### 2.1. Materials

A white Portland cement: CEM I 52.5 N CE CP2 NF Calcia was used. The specific density is 3.15. The used sand in the mortars is standard sand certified CEN, EN 196-1 provided by the Société Nouvelle du Littoral, located at Leucate, France.

**Figure 1:**
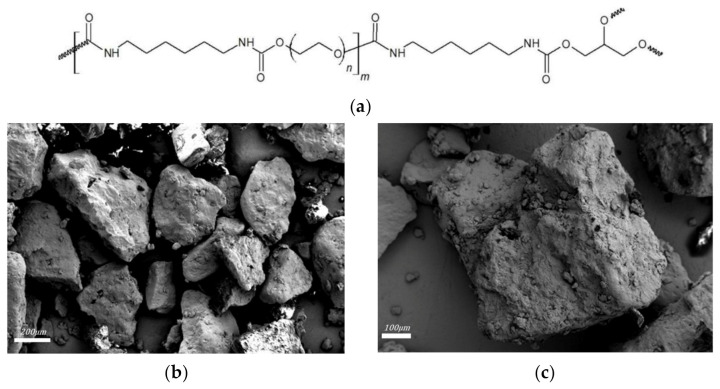
(**a**) Chemical structure of the homemade s/s PCMs PUX1520; (**b**,**c**) SEM images of s/s PCMs grains.

Poly (ether urethane) based s/s PCMs are homemade PCMs produced by following synthesis process of PUX1520 patented by Harlé et al. [36]. PEG (Mn = 1500 g·mol^−1^) and glycerol were purchased from Carl Roth GmbH+Co and hexamethylene diisocyanate (HMDI) from VWR. Synthesis reaction occurred in bulk under argon atmosphere at 60 °C during 45 min in a reactor with controlled stirring. Post-curing was carried out at 100 °C for 4 h in an oven. Blocks of polymers were finely ground using a Knife Mill grindomix GM200 from Retsch^®^ The s/s PCMs used in this study present an equivalent diameter ranging from 300 to 600 µm; the size was controlled by sieving. Chemical structure and grain images of PUX1520 are presented in Figure 1. Scanning Electron Microscopy images were obtained at IMAT–CY University with a ZEISS GeminiSEM 300.

### 2.2. Sample Preparation

A known amount of s/s PCMs grains was dispersed in dichloromethane for 30 min under stirring. Mixtures were poured in cellulose extraction cartridge of 5 µm pores. The solution flowed dropwise through the cartridge was evaporated giving soluble part of PCMs. Mass of resulting PCMs and initial PCMs were compared leading to a PCMs soluble proportion of 56 wt%.

Thus, when PCMs are added to the formulation, only the insoluble part is used to compute the solid volume fraction. This varies from 0 to 14.8% (Equations (1) and (2)). The addition of PCMs also increases the concentration of soluble PCM in the water of the formulations. It increases from 0 to 112 10^−3^ mol·L^−1^.

(1) $$\varphi =\frac{\;\frac{m_{PCM}}{\rho_{PCM}}\times 0.44}{\;\frac{m_{PCM}}{\rho_{PCM}}+\frac{m_{water}}{\rho_{water}}+\frac{m_{cem}}{\rho_{cem}}}$$

(2) $$\varphi =\frac{\;\frac{m_{PCM}}{\rho_{PCM}}\times 0.44}{\frac{m_{cem}}{\rho_{cem}}+\frac{m_{PCM}}{\rho_{PCM}}+\frac{m_{sand}}{\rho_{sand}}+\frac{m_{water}}{\rho_{water}}}$$

With $m_{PCM}$, the mass of s/s PCMs (g), $\rho_{PCM}$ the density of s/sPCMs, $m_{water}$ the mass of water (g), $\rho_{water}$, the density of water, $m_{cem}$ the mass of cement (g), $\rho_{cem}$ the density of cement, $m_{sand}$ the mass of sand (g) and $\rho_{sand}$ the density of sand.

The cement pastes were prepared from a premix previously prepared with 450 g of dry cement plus various amount of s/s PCMs. The different mix designs, see Table 1 for cement pastes and Table 2 for mortars, were firstly mixed during 60 s at 62 rpm with 225 g of water in a planetary mixer. The pastes were then mixed by hand to ensure that no dry particles of cement or s/s PCMs remained on the mixer wall. Finally, in order to obtain a homogeneous material, the mixtures (cement pastes and then mortars) were stirred during 90 s. All cement and mortar suspensions were prepared with a 0.5 water to cement mass ratio. The quantity of s/s PCMs ranged from 0 to 2.2 10^−2^ mol·L^−1^. Neither sedimentation nor bleeding was observed after the sample preparation procedure.

### 2.3. Rheological Measurements

**Figure 2:**
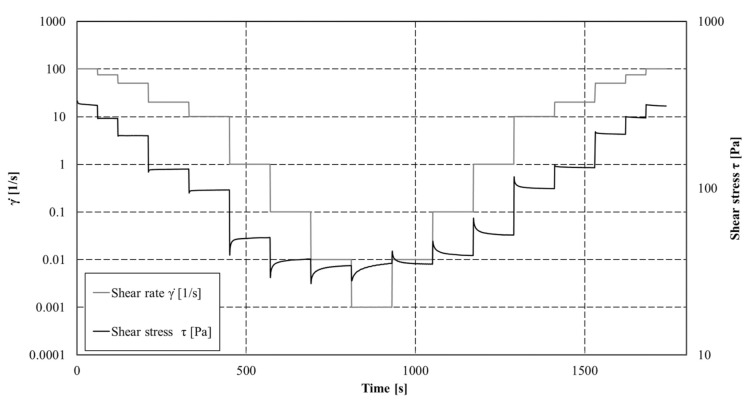
Shear rate applied and shear stress measured as function of time (example on a cement paste with 225 g of PCMs).

Rheological measurements were performed using a rheometer (Anton Paar MCR 102^®^) equipped with a four-bladed vane (H_v_ = 4 cm; R_v_ = 1.1 cm; R_c_ = 4.51 cm) and cylindrical cup. The temperature was controlled and fixed to 20 °C using a Peltier temperature control device located around the cup. Experiments were conducted with controlled shear rate measurements by applying first a restructuration protocol (shear rate decreasing) followed by a destructuration protocol (shear rate increasing). The retained shear rate values are given as follow: 100, 75, 50, 20, 10, 1, 0.1, 0.01, and 0.001 s^−1^. Each set point was fixed during at least 90 s to ensure the stabilisation of the shear stress value. Each suspension and solution was characterized at least twice to ensure the reliability of the measurements. For each step, shear stress (*τ* (Pa)) was measured each second and the steps last until a steady state. Mean shear stresses were obtained from these steady states and flow curves could then be analysed. An example of applied shear rates and measured shear stress is given in Figure 2.

### 2.4. Spectrophotometer

**Figure 3:**
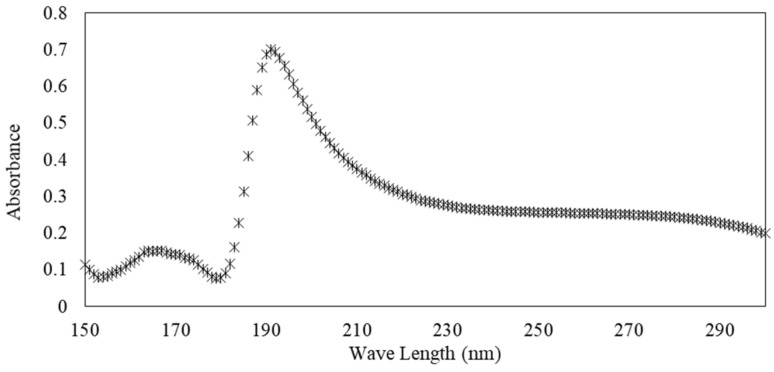
Illustration of the UV absorbance of solution of s/s PCMs.

UV–visible absorbance measurements were performed using a spectrophotometer Uvikson XS double beam with a wavelength precision of 0.3 nm. Spectral range from ʎ = 150 to 300 nm was used and the measurement time was 0.1 s. Solutions were poured into UV quartz cells with 10 mm path length. Figure 3 illustrates the evolution of the absorbance as a function of the wavelength for an s/s PCMs/water solution (10^−1^ g·L^−1^).

**Figure 4:**
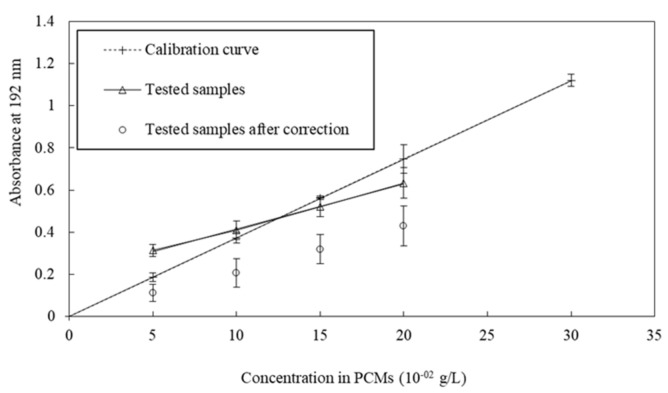
Comparison of tested samples with calibration curve.

We measured the evolution of the maximum absorbance as a function of the concentration of s/s PCMs by displaying a linear correlation between concentration and absorbance (Figure 4). This result points out the possibility to use absorbance measurement in order to determine the s/s PCMs concentration in solution and to deduce the amount trapped in the cement paste.

Therefore, the liquid part of the cement/water or the cement/s/s PCMs/water mixtures were analysed by means of UV–visible absorbance measurements following this procedure: cement was mixed with different amounts of s/s PCMs powder and water (water/cement mass ratio = 0.5). The mixtures were poured into 15 mL Falcon tubes. The tubes were centrifuged for 10 min at 496 rpm. Extracted liquids were once again centrifuged for 10 min at 496 rpm. The newly extracted liquids were diluted into distilled water (1/1000) and analysed with the spectrophotometer UV-visible. In order to perform calibration curves, known amounts of s/s PCMs were added to extracted liquids of 0% s/s PCMs cement paste before dilution. Triplicates of calibration curves and tested samples were carried out.

## 3. Results and Discussion

### 3.1. Assessment of s/s PCMs Trapped in Cement Matrix

In this study, we use s/s PCMs based on cross-linked poly(ether urethane) (PUX)s. This material is partially soluble in water with a soluble part of 56 wt%. In order to understand the rheological behaviour of the composites, it is relevant to understand the interactions between cement grains and s/s PCMs. The measurement of trapped s/s PCMs in cement matrix allows knowing the part of soluble s/s PCMs adsorbed on cement grains.

As the centrifugation extracts s/s PCMs solution, i.e., the interstitial fluid of the cement paste, we measure the concentration by comparing the final concentration of this solution to the initial concentration. It then allows computing the relative amount of s/s PCMs that can be trapped in the cement matrix.

Samples with four different amounts of s/s PCMs (5, 10, 15 and 20 × 10^−2^ g·L^−1^) in cement paste were analysed. UV absorbance of their interstitial fluid is plotted in Figure 4 as a function of the initial content of s/s PCMs introduced in the cement paste. Linear regression (correlation coefficient = 0.999) of s/s PCMs/cement paste samples shows the –y-intercept different from zero. This implies that a correction must be applied to the absorption values of tested samples. This correction is required to compensate for the lack of water available to solubilize the s/s PCMs due to the competition between the cement hydration and the s/s PCMs solubilization. The intercept was subtracted from absorption values of the tested values thus giving corrected values.

The corrected values of tested samples were compared to the calibration curve leading to the amount of trapped s/s PCMs on cement following Equation (3):

(3) $$\mathrm{Tr}=\frac{C_{i}-\frac{\mathrm{Ab}}{s}}{C_{i}},$$

With Tr, the relative amount of trapped s/s PCMs on cement, C_i_ the initial content in s/s PCMs of the cement paste tested (g·L^−1^), Ab the corrected value of absorbance, s the slope value of the calibration curve (L·g^−1^).

Table 3 displays the relative amount of s/s PCMs trapped in cement according to the initial content. The mean value of trapped s/s PCMs is 42%; this value includes s/s PCMs adsorbed on cement grain and non-soluble s/s PCMs embedded in the cement matrix. Assuming that all non-soluble part of s/s PCMs, i.e., 44% of s/s PCMs is embedded in cement paste which means that there is no absorption at all.

As the s/s PCMs used are protected by a patent [36], we cannot compare directly the results with those of the literature. Nevertheless, Bessaies-Bey et al. ([37]) studied the adsorption of three PEGs of different molar masses on the cement surface. They reported that PEGs are not adsorbed by the mineral surfaces, but remain in the interstitial solution. This is consistent with our results as the s/s PCMs used in this study are mainly composed of PEG.

### 3.2. Polymer Suspension

**Figure 5:**
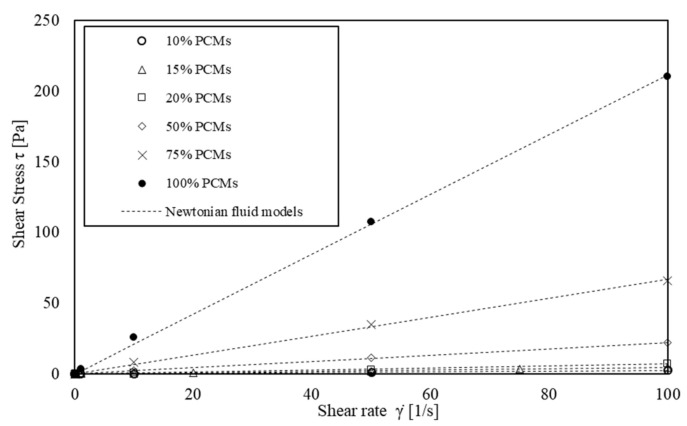
Shear flow curves of the interstitial fluid: PCMs in water.

The rheological properties of six solutions of s/s PCMs and water (concentration ranging from 5.60 × 10^−3^ to 56.0 × 10^−3^ mol·L^−1^) were analysed. The average flow curves are shown in Figure 5. The rheological behaviour is well modelled by a Newtonian viscous law, Equation (4):

(4) $$\tau =\eta \;\dot{\gamma }$$

With *τ*, the shear stress (Pa); *η*, the viscosity (Pa·s) and $\dot{\gamma }$, the shear rate (s^−1^). The best-fitting allows for modelling the experimental data with correlation coefficients higher than 0.99 (Figure 5).

Table 4 shows the deduced viscosity. In the following part, the effect of s/s PCMs solubilization combined to solid inclusions sheared in the suspension is included in a viscosity model.

The relative viscosity can be related to the properties of polymers, i.e., the conformation for a semi-diluted regime such as the one used in this study. The viscosity of a soluble polymer in an aqueous solution is commonly defined by the polynomial Huggins Equation (5):

(5) $$\eta =\eta_{0}+\eta_{0}\left[\eta \right]c+\eta_{0}{\left[\eta \right]}^{2}c^{2}k$$

With *η* the viscosity (Pa·s), *η*_0_ the viscosity of interstitial fluid (Pa·s), [*η*] the intrinsic viscosity (L·mol^−1^) related to a single molecule in solution, *c* the concentration of the solution and *k* a dimensionless parameter called Huggins constant.

Kirincic and Klofutar [38] reported values of [*η*] and *k* for PEG regarding the molar mass of the polymer. They found that molar mass values range from 1500 g·mol^−1^ to 35,000 g·mol^−1^ and values of [*η*] range from 11 to 2000 (L·mol^−1^). Since the s/s PCMs are mainly composed of PEG, it is assumed that the [*η*] must take its value in the same range. It is also reported that *k* generally takes its value between 0.3 and 0.5 [39].

However, in the suspension, an insoluble part of the polymer may have an effect on the evolution of the viscosity through a structuration mechanism. The insoluble part can be considered as a solid inclusion in a suspension whose model is generally well defined by the Krieger–Dougherty Equation (6) [40]

(6) $$\eta =\eta_{0}{\left(1-\frac{\Phi }{\Phi_{m}}\right)}^{-\left[\eta \right]\Phi_{m}}$$

where *η* is the plastic viscosity of the suspension (Pa·s), *η*_0_ the plastic viscosity of the interstitial fluid (Pa·s), *Φ* the volume fraction of the solid, $\Phi_{m}$ the maximum volume fraction and [*η*] the intrinsic viscosity of the solid.

Here, [*η*] is different from that of the Huggins equation since it refers to a single solid particle and its value depends on its shape. Barnes et al., [41] and Struble et al., [42] reported that for spherical monodisperse particles, the solid volume fraction $\Phi_{m}$ range from 0.6 to 0.7 and [*η*] is equal to 2.5. However, they also established that with an asymmetry of the particles *φ_m_* can be lower than 0.6 and with high polydispersity, it can be higher than 0.7. As for [*η*], they reported that for angular particles, it could take its values up to 5. Please note that according to Figure 1, the s/s PCMs grains cannot be considered spherical.

Thus, the mixing between the insoluble and soluble parts implies that the interstitial fluid of the suspension presents a viscosity, which depends on the s/s PCMs content. A law is then proposed combining both the viscosity of the polymer in solution and the viscosity of the granular Suspension (7):

(7) $$\eta =\left(\eta_{0}+\eta_{0}{\left[\eta \right]}_{1}c+\eta_{0}{\left[\eta \right]}_{1}{}^{2}c^{2}k\;\right){\left(1-\frac{\Phi }{\Phi_{m}}\right)}^{-{\left[\eta \right]}_{2}\Phi_{m}}$$

**Figure 6:**
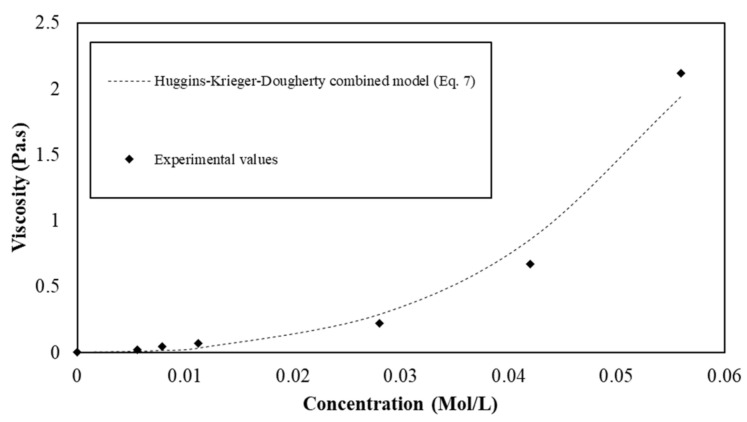
Viscosity of the s/s PCMs water suspension as a function of the s/s PCMs soluble part concentration.

The proposed equation is used to model the experimental viscosity values from measurements (Figure 6). The parameters ${\left[\eta \right]}_{1}$, k, $\Phi_{m}$ and ${\left[\eta \right]}_{2}$ are computed to fit the experimental data and their values are reported in Table 5. We observe from Figure 6 that the equation provides a suitable model to describe the viscosity of suspensions with semi-soluble s/s PCMs in water.

### 3.3. Effect of the s/s PCMs on the Structuration of the Flow

Several laws (modified Bingham, Hershel–Bulkley, Ellis, Casson or Eyring) were proposed to model the rheological behaviour of cement pastes. Herschel–Bulkley (HB) is used in this study to well describe the viscoplastic rheological behaviour of the suspensions including the possible structuration under shearing (Equation (8)) [43]:

(8) $$\tau =\tau_{0}+k{\dot{\gamma }}^{n}$$

where *τ* (Pa) is the shear stress, $\dot{\gamma }$ (s^−1^), the shear rate, *τ*_0_ (Pa) the yield stress, *n* the flow behaviour index and *k* the consistency (Pa·s*^n^*).

**Figure 7:**
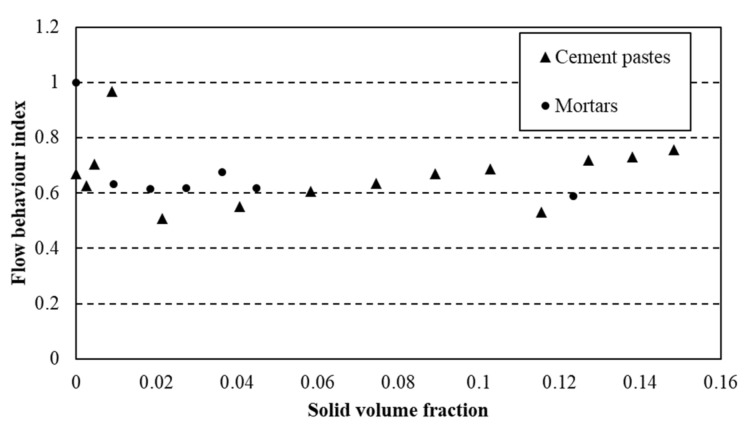
Flow behaviour index *n* for cement pastes and mortars.

This law was used to describe flow curves of cement pastes and mortars and the parameters *τ*_0_, *k* and *n* were computed to fit the experimental values from rheological measurements. The flow behaviour index for cement pastes and mortars is plotted as a function of solid volume fraction (relative to s/s PCMs content) (Figure 7).

The index of flow behaviour does not evolve as a function of the amount of s/s PCMs in the suspension. Moreover, the addition of sand in the paste does not change the value of this parameter. Therefore, it is relevant to observe that only the cementitious particles rule the shear-thinning behaviour of the mixtures under shearing. A mean value of 0.65 of this index value is computed. This value is used to model flow curves by the HB model and the values of $k{\dot{\gamma }}^{n}$ for $\dot{\gamma }=0.1;10\;\mathrm{and}\;100$ are compared to those of free *n* index (i.e., from fitting) flow curves.

**Figure 8:**
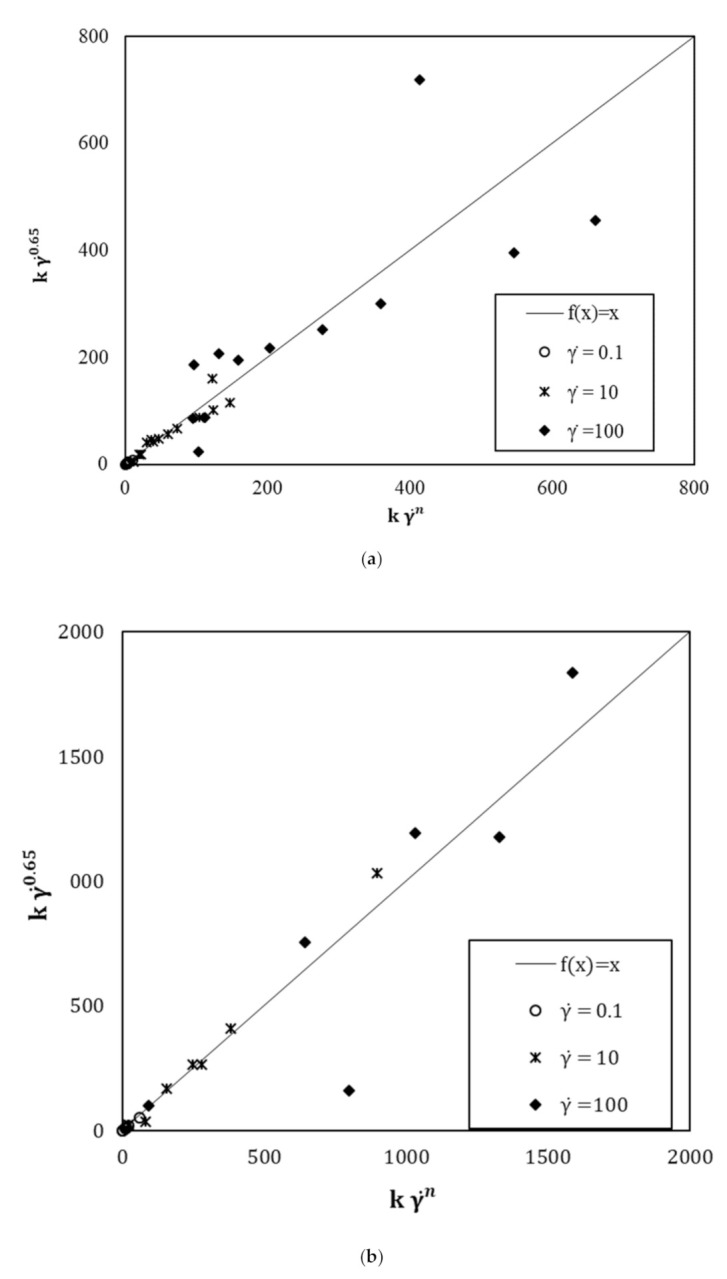
Comparison of $k{\dot{\gamma }}^{0.65}$ with $k{\dot{\gamma }}^{n}$ for (**a**) cement pastes and (**b**) mortar pastes.

Figure 8 displays the divergence between the shear stress values when *n* is freely computed and when the fixed value of *n* is 0.65. It is observed that there is no or little divergence for low shear gradients. On the other hand, it is found that a divergence occurs for high shear gradients. However, shear rates higher than 50 s^−1^ are not representative of the real implementation conditions in the construction process where the suspension is commonly poured by gravitational effects. Moreover, the flow curves modelled with an *n* fixed value of 0.65 show a correlation coefficient with the experimental values greater than 0.99. Thus, it can be assumed that 0.65 is a reliable parameter to model the rheological behaviour of cement and mortars containing s/s PCMs.

### 3.4. Effect of the s/s PCMs on the Viscosity

**Figure 9:**
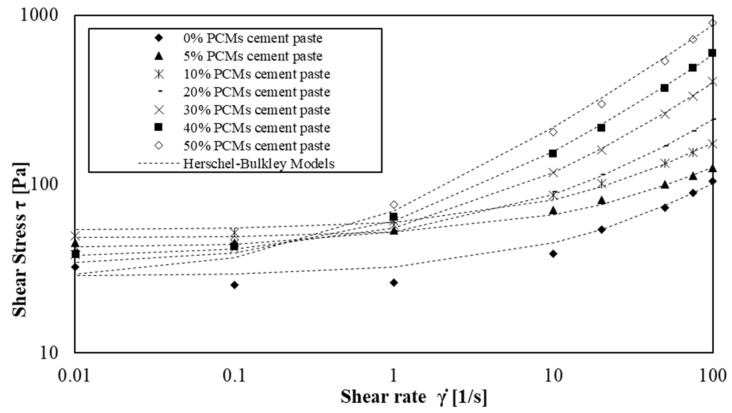
Shear flow curves of cement paste with amount of s/s PCMs ranging 0 wt% to 50 wt%.

**Figure 10:**
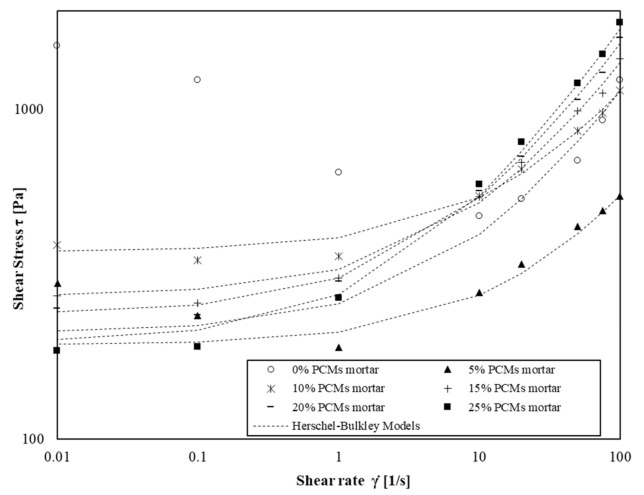
Shear flow curves of mortars with amount of s/s PCMs ranging 0 wt% to 75 wt%.

We then compare the experimental measurements with the HB model using a value of index exponent of 0.65. Figure 9 and Figure 10 illustrate the shear flow curves of cement pastes and mortar pastes containing s/s PCMs and the model using a fixed value of *n* = 0.65.

We observe that Herschel–Bulkley models fit the experimental values for the four shear rate decades except for mortars without and with a low amount of s/s PCM. In this case, the flow curves are only well modelled for shear rate values above 1 s^−1^. This phenomenon occurs particularly when there is no or low amount of s/s PCMs in mortars suspension. This result indicates that s/s PCMs could decrease the colloidal interactions of cement grains.

**Figure 11:**
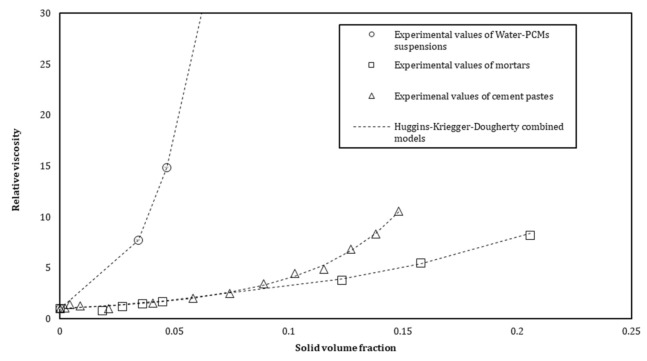
Relative viscosity as function of the solid volume fraction of s/s PCMs for cement pastes and mortars.

The relative viscosity evolution as a function of the solid volume fraction of s/s PCMs is shown in Figure 11 for the three distinct suspensions (mortar, cement and modified water). We observe from Figure 11 that the s/s PCMs have an influence on the relative viscosity of cement pastes and mortars. As observed previously, a structuration mechanism cannot be expressed only by the effect of solid inclusions. A combined Huggins and Krieger–Dougherty equation seems to be more appropriate to describe the evolution of the relative viscosity of a semi-soluble polymer solution. For cement pastes and mortars, cement and sand are implemented in the equation to compute the solid volume fraction. The relative viscosity is then described by the following Equation (9):

(9) $$\frac{\eta }{\eta_{0}}=\left(1+{\left[\eta \right]}_{1}c+{\left[\eta \right]}_{1}{}^{2}c^{2}k\;\right){\left(1-\frac{\Phi }{\Phi_{m}}\right)}^{-{\left[\eta \right]}_{2}\Phi_{m}}$$

Values computed for cement pastes are similar or very close to that of the s/s PCMs solution in water (Table 5). The only intrinsic viscosity of soluble polymer evolves differently which means that cement could generate a slight modification in the conformation of soluble polymer chains. Clarification of the role of s/s PCMs, when included in water, helps us to better highlight its effect in the cement paste and finally in the mortar. Volumes of s/s PCMs, which were considered in the study, clearly dominate the viscosity structuration of the different scales of suspensions.

We also observe in Table 5 that the values of ${\left[\eta \right]}_{2}$ of mortars are slightly different from the cement pastes and the interstitial solution. The decrease in the value of the power exponent ${\left[\eta \right]}_{2}$ means that the sand particles could be in excess regarding the amount of binder and therefore affects the values of the fitting coefficient. This exponent, relative to the shape of the inclusion, is 2.5 for spherical particles [42]. The shape of s/s PCMs should not be affected by the incorporation of sand in the mixture and yet the model cannot be fitted with ${\left[\eta \right]}_{2}$ equal to five as in the case of s/s PCMs suspension in water and s/s PCMs in cement paste.

The variations of the Huggins constant *k* were previously studied [39,44,45]. This parameter is reported to be solvent and temperature-dependent. Although this value can be constant for a solvent–polymer system, it may also be affected by variations of the shear rate. It could be assumed that sand content modifies the local hydrodynamic interactions of suspensions containing s/s PCMs. Adding a certain amount of binder or cement paste should help to maintain the solid inclusions as inert particles that do not rule the rheological behaviour of the fluid. Thus, the sand plays a role in the rheology of the cementitious material containing s/s PCMs. Nevertheless, further research is required to clarify the interaction of the sand particles with the s/s PCMs.

### 3.5. Yield Stress Evolution as a Function of the s/s PCMs Amount

**Figure 12:**
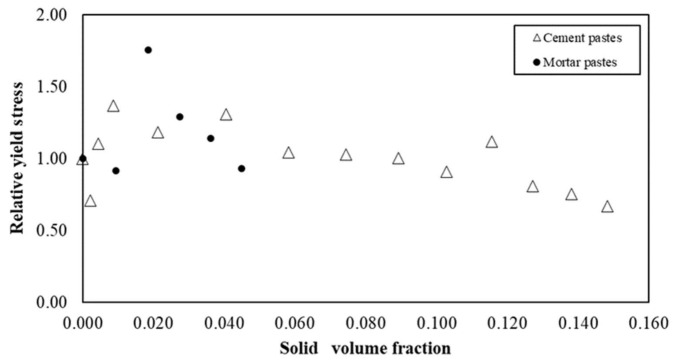
Relative yield stress for cement and mortar pastes with s/s PCMs.

Figure 12 illustrates the relative yield stress versus the solid volume fraction of s/s PCMs in cementitious and mortar suspensions. In both cases and more specifically for cement pastes, yield stress values appear to be constant regardless of the content of s/s PCMs. This result differs from that generally reported in the literature. Indeed, increasing the quantity of solid inclusions generally leads to an increase in yield stress in an exponential law [46,47]. Here, the results suggest that a lubrication phenomenon acts in competition with the physico-chemical interactions that generate the yield stress.

## 4. Conclusions

In this work, the influence of granular poly (ether urethane)-based s/s PCMs on the rheology of cement and mortar suspensions was studied. Rheological measurements of s/s PCMs suspension in water revealed that a combined Huggins and Krieger–Dougherty model is reliable to describe the viscosity structuration induced by the s/s PCMs content. This model aims to describe in detail the increasing viscosity with an increasing amount of s/s PCMs. Such behaviour allows for explaining the semi-soluble effect of the s/s PCMs.

The visco-plastic behaviour exhibited by the analysed cement pastes and mortars is well described by the Herschel–Bulkley model. The shear thinning behaviour of the mineral suspensions is highlighted and a value of the structuration index, which does not depend on the amount of s/s PCMs, is adapted to describe the flow curves. The modified Huggins–Krieger–Dougherty model seems to be well adapted to explain the relative viscosity increasing induced by the incorporation of s/s PCMs in the cement pastes and then in the mortars. While the water slurry and cement paste have similar parameters to the Huggins–Krieger–Dougherty model, the inclusion of sand in the case of mortar affects these parameters. Further research is needed to fully understand this phenomenon. Finally, the present experimental investigation shows that the computed limit shear stress is not affected by adding the s/s PCMs.

## Figures and Tables

**Table 1:** Mix design of cement pastes.

Cement Paste					
Cement (g)	Water (g)	PCMs			
		Mass (g)	%PCMs	Concentration of the Soluble Part (10^−3^ mol·L^−1^)	*Φ* (10^−3^ Vol sol/Vol tot) ^1^
450	225	0	0	0.0	0.0
450	225	2.5	0.5	0.5	2.22
450	225	4.5	1	1.12	4.43
450	225	9	2	2.24	8.77
450	225	22.5	5	5.6	21.3
450	225	45	10	11.2	40.6
450	225	67.5	15	16.8	58.2
450	225	90	20	22.4	74.4
450	225	112.5	25	28	89.2
450	225	135	30	33.6	102.9
450	225	157.5	35	39.2	115.5
450	225	180	40	44.8	127.2
450	225	202.5	45	50.4	138.1
450	225	225	50	112	148.3

^1^ Solid volume fraction described in Equation (1).

**Table 2:** Mix design of mortars.

Mortar						
Cement (g)	Sand (g)	Water (g)	PCMs			
			Mass (g)	% PCMs	Concentration of the Soluble Part (10^−3^ mol/L)	*Φ* (10^−3^ Vol sol/Vol tot) ^1^
450	1350	225	0	0	0.0	0.0
450	1350	225	22.5	5	5.6	9.2
450	1350	225	45	10	11.2	18.0
450	1350	225	67.5	15	16.8	27.4
450	1350	225	90	20	22.4	36.2
450	1350	225	112.5	25	28	44.8
450	1350	225	337.5	75	84	123

^1^ Solid volume fraction described in Equation (2).

**Table 3:** Amount of trapped s/s PCMs.

C_i_ (10^−2^ g·L^−1^)	Tr (%)
5	40.39 ± 9
10	44.40 ± 9
15	42.99 ± 8
20	42.40 ± 8

**Table 4:** Viscosity of s/s PCMs in water solution.

PCMs/Water (wt/wt)	Concentration of the Soluble Part (10^−3^ mol·L^−1^)	*Φ* (10^−2^ Vol sol/Vol tot)	Viscosity (10^−3^ Pa·s)
0%	0	0	1.0
10%	5.60	3.44	23.9
15%	7.84	4.67	44.2
20%	11.2	6.38	71.8
50%	28.0	13.1	223
75%	42.0	17.1	670
100%	56.0	20.2	2116

**Table 5:** Huggins–Krieger–Dougherty combined model parameters.

	${\left[\eta \right]}_{1}$ (L·mol^−1^)	*k*	$\Phi_{m}$	${\left[\eta \right]}_{2}$	Correlation Coefficient (R^2^)
Suspensions of PCMs	25.5	0.4	0.5	5	0.98
Cement pastes	24	0.4	0.5	5	0.99
Mortar pastes	20	0.015	0.5	2.5	0.99

## Data Availability

The data presented in this study are available on request from the corresponding author.
